# Temporal and Fronto-Central Auditory Evoked Responses in Children with Neurodevelopmental Disorders: A Scoping Review

**DOI:** 10.3390/neurosci5040048

**Published:** 2024-12-10

**Authors:** Zohreh Ahmadi, Fauve Duquette-Laplante, Shanna Kousaie, Benjamin Rich Zendel, Amineh Koravand

**Affiliations:** 1 Audiology Program, School of Rehabilitation Sciences, Faculty of Health Sciences, University of Ottawa, Ottawa, ON K1S 5L5, Canada; zahma083@uottawa.ca (Z.A.);; 2 School of Psychology, Faculty of Social Sciences, University of Ottawa, Ottawa, ON K1N 6N5, Canada; 3 Faculty of Medicine, Memorial University of Newfoundland, St. John’s, NL A1B 3V6, Canada

**Keywords:** auditory evoked potentials, T-complex, primary auditory area, secondary auditory area

## Abstract

At the cortical level, the central auditory neural system (CANS) includes primary and secondary areas. So far, much research has focused on recording fronto-central auditory evoked potentials/responses (P1-N1-P2), originating mainly from the primary auditory areas, to explore the neural processing in the auditory cortex. However, less is known about the secondary auditory areas. This review aimed to investigate and compare fronto-central and T-complex responses in populations at risk of auditory dysfunction, such as individuals with neurodevelopmental disorders. After searching the electronic databases (PubMed, Web of Science, Scopus, and Ovid), ten studies encompassing six neurodevelopmental disorders were included for the analysis. All experimental populations had atypical T-complexes, manifesting as an absence of evoked responses, shorter latency, and/or smaller amplitude. Moreover, in two experimental groups, dyslexia and attention deficit/hyperactivity disorder (ADHD), abnormal T-complex responses were observed despite the presence of normal fronto-central responses. The presence of abnormal T-complex responses in combination with normal fronto-central responses in the same population, using the same experiment, may highlight the advantage of the T-complex for indexing deficits in distinct auditory processes or regions, which the fronto-central response may not track.

## 1. Introduction

**Figure 1:**
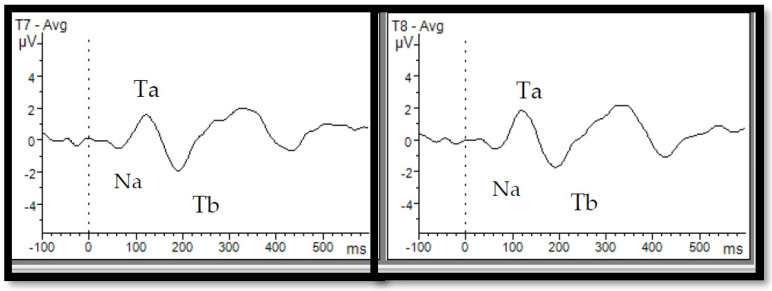
Auditory evoked potentials from the temporal lobe and T-complex. T-complex waveforms—consisting of successive Na, Ta, and Tb peaks—were recorded at the left (T7) and right (T8) temporal sites in adults in response to a 1 kHz tone. This figure was derived from data collected at our Electrophysiology Lab, University of Ottawa, and has not been published before.

Listening difficulties are commonly reported in children with neurodevelopmental disorders. In many cases, these challenges are not fully explained by disordered transduction in the cochlea or abnormal early processing along the auditory brainstem pathway. It is therefore likely that these listening challenges are associated with how auditory information is processed in the cortex. Auditory information enters the cortex through primary auditory areas and then projects to secondary and tertiary auditory areas [1,2]. The primary auditory cortex (PAC) is the first cortical region involved in acoustical processing and receives inputs from the thalamus and the medial geniculate body (MGB) [3,4]. The PAC can be segmented into three regions; from anterior to posterior, these regions consist of the planum polare (PP), Heschl’s gyrus (HG), and the planum temporale (PT) (Figure 1). HG is located on the supratemporal plane, extending diagonally from the superior temporal gyrus (STG) [5,6]. The STG encompasses the secondary auditory areas and anatomically interfaces with the lower-level auditory areas and higher-level structures contributing to language, music, and other forms of auditory processing [7]. The STG plays a special role in the neural analysis of speech sounds and phonological processing [8,9].

Impairments in the central auditory system may lead to poor listening abilities, mostly labeled as auditory processing disorder (APD) [10]. This disorder affects around 5–7% of children [11,12,13,14]. APD and other neurodevelopmental disorders such as language impairment (LI), attention deficit/hyperactivity disorder (ADHD), and autism share a common characteristic: developmental delays, which can impair personal, social, academic, or occupational functioning [15]. Moreover, various developmental disorders may overlap with each other [16]. Multiple studies have shown that LI, ADHD, and autism are often comorbid with APD [16,17,18,19]. Hence, evaluating auditory processing, along with language, attention, and other cognitive abilities, in these populations is highly recommended [20]. Over the years, auditory evoked potentials (AEPs) have been utilized to explore impairments in the auditory cortex in these populations [21,22,23,24,25].

AEPs are series of changes in electrical brain activity triggered by acoustic stimulation. At the cortical level, there are two categories of AEPs: fronto-central and temporal responses [26,27]. Fronto-central responses include P1-N1-P2-N2 and are measured over fronto-central sites but arise mainly from primary auditory areas and index the acoustical processing of sound [27,28]. Numerous studies recording P1-N1-P2-N2 have indicated deficits in cortical auditory processing among individuals with neurodevelopmental disorders [22,29,30,31,32]. For instance, in a group of children with language impairments, smaller amplitudes and/or longer latencies of P1, N1, and P2 were observed compared to those of responses obtained from typically developing (TD) groups [29,33].

The T-complex, or temporal auditory response, is another AEP that corresponds to fronto-central responses in terms of timing but is recorded over temporal sites and consists of the Na-Ta-Tb sequence (Figure 1) [26,34,35]. Both fronto-central and temporal responses are sensitive to physical modulations of stimuli such as changes in intensity, interstimulus interval (ISI), and stimulus onset asynchrony (SOA) in children and adults [36,37,38,39]. For instance, consistent with fronto-central responses, the T-complex’s amplitude increases with a longer SOA [40]. Despite sharing similar time windows, there are some differences between T-complex and fronto-central responses. In fact, P1-N1-P2 has a frontal topography, represents activity mainly in the primary auditory areas [41,42,43], and features a tangential dipole orientation [26,41,42]. In contrast, the T-complex has a temporal topography and reflects the activity of lateral temporal structures within the posterior lateral superior temporal gyrus, with a radial dipole orientation [26,35,41]. Maturational studies also suggest that the T-complex matures earlier than the fronto-central response (about 4–5 years old vs. 8–9 years old) and remains stable with increasing age [35,44,45]. Considering the developmental trajectory, it is thought that the T-complex relates to the more complex processing of sound and better correlates with speech and language processing as compared to fronto-central responses [40,44,46].

Multiple studies have demonstrated the effectiveness and reliability of the T-complex in assessing auditory processing at the cortical level [21,45,47]. The evaluation of fronto-central and T-complex waveforms in individuals with auditory dysfunction is promising, as it can yield information regarding the origin of the deficit, specifically, whether it arises from within the superior temporal plane or the posterior lateral superior temporal gyrus [47].

To the best of our knowledge, no review has investigated and compared the properties of the T-complex and fronto-central responses of different populations with auditory dysfunctions. Therefore, this review aims to compile studies measuring T-complex and fronto-central responses in populations with neurodevelopmental disorders. Specifically, the first objective is to report and document the properties of T-complex responses in populations with neurodevelopmental disorders. The second objective is to explore the similarities and differences between the T-complex and fronto-central responses.

## 2. Methods

A scoping review was carried out in order to incorporate multiple types of studies with disparate methodologies [48]. Two reviewers (ZA and AK) were involved in the review process [49]. The second reviewer (AK) analyzed only articles included by the first reviewer, using a liberal accelerated approach. If a disagreement arose, a third reviewer was tasked with its resolution (FDL) [49].

In order to avoid missing any related articles, key words were selected in a manner designed to provide as many as studies as possible on the T-complex, with no preference for age or population characteristics. Then, the studies matched with the target population (neurodevelopmental disorder) were identified during the screening steps based on the inclusion criteria. The search terms relating to the T-complex included auditory or speech-evoked potential or response, electroencephalography (EEG) assessment, electrophysiological indices, neural encoding, cortical processing, neural activity, event-related potential (ERP) response, neural response, mismatch negativity, speech signal, oscillatory EEG response, CAEP, auditory neural integrity, event-related potential, auditory measure, electrophysiological measure, and electroencephalography.

Databases, including PubMed, the Web of Science, Scopus, and Ovid, were searched separately, and results were compiled in the Covidence database, where search strategies were dated and organized. Conference papers, master’s dissertations, and doctoral theses (gray literature) were searched through ProQuest Dissertations and Theses Global, Google Scholar, and Theses Canada.

This review included literature published until 2023. The method for screening and finding the related studies followed the PRISMA process [50]. In the initial screening, reviewer 1 (ZA) retained or rejected articles based only on title analysis. Both reviewers (ZA and AK) completed the second and third screenings; in these steps, the eligibility was based on abstract and full-text analyses, respectively. The exclusion and inclusion criteria are described in Appendix A. According to the goals of this review, only studies with participants with neurodevelopmental disorders were included. Also, because using a translator was not feasible for this study, only articles in English were selected.

## 3. Results

**Figure 2:**
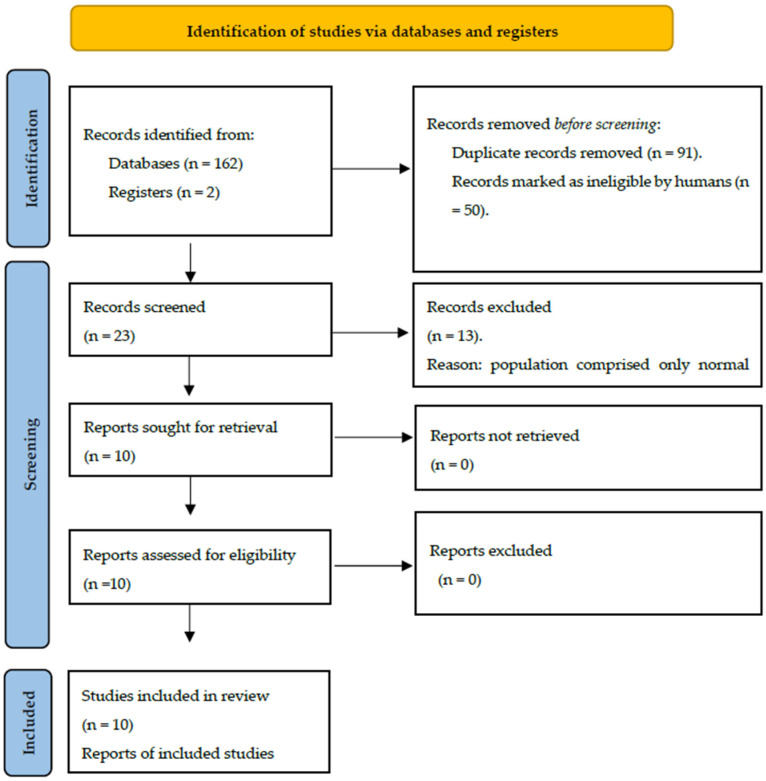
Flow chart for the scoping review process [50].

From the databases, a total of 164 articles were identified. Following the initial screening step and removal of duplicates (*n* = 91), 73 articles remained. Among the 73 articles, 23 articles were included based on title evaluation (Bishop et al., 2012; Bruneau et al., 2003; Bruneau et al., 1999; Bruneau et al., 2015; Cacace et al., 1988; Carrillo-de-la-Peña, 1999; Čeponien et al., 1998; Clunies-Ross et al., 2015; Clunies-Ross et al., 2018; Gomes et al., 2012; Groen et al., 2008; J. A. Hämäläinen et al., 2011; Ponton et al., 2002; Shafer et al., 2011; Silva et al., 2020; Rinker et al., 2021; Taylor et al., 2003; Tonnquist-Uhlén, 1996; Tonnquist-Uhlen et al., 2003; Wolpaw & Penry, 1975, 1977; Wagner et al., 2016; Woldorff & Hillyard, 1991) [18,21,23,26,29,35,36,37,39,40,45,46,47,51,52,53,54,55,56,57,58,59,60]. As a result of abstract screening, 13 studies were excluded, as they did not include abnormal populations among their participants (Bruneau et al., 2015; Cacace et al., 1988; Carrillo-de-la-Peña, 1999; Čeponien et al., 1998; Clunies-Ross et al., 2015; Clunies-Ross et al., 2018; Ponton et al., 2002; Silva et al., 2020; Tonnquist-Uhlen et al., 2003; Wolpaw & Penry, 1975, 1977; Wagner et al., 2016; Woldorff & Hillyard, 1991) [26,35,36,37,40,45,46,47,51,52,56,59,60]. The remaining ten articles underwent a thorough full-text screening, and all of them met the eligibility criteria and were consequently included in this study. The PRISMA flow diagram is presented in Figure 2. The data, encompassing information about experimental and control groups, event-related potentials (ERPs) of interest, and the results, have been succinctly summarized in Table 1.

### 3.1. Study Characteristics

#### 3.1.1. Type of Disorder

Four studies were found to assess LI using the T-complex [23,29,55,57]. A further study assessed ADHD [53]; two assessed dyslexia [39,58]; one assessed Down syndrome (DS) [54]; and two assessed autism [18,21]. These neurodevelopmental disorders often have comorbidity with APD, potentially sharing symptoms related to auditory dysfunction [16,61]. Notably, there was no study investigating the T-complex in children diagnosed with auditory processing disorder.

#### 3.1.2. Age

Only one study examined the T-complex in adults, while the remaining studies focused on child participants. One study included both children (aged 7–12 years) and teenagers (aged 13–16 years) [29]. The age range varied from the youngest participant at 4 years old to the oldest teenager at 16 years old [29]. However, among the included studies, one study was conducted on adults aged between 18 and 45 years old (average age was 25 years old).

#### 3.1.3. Stimuli

Different stimuli were used in the included studies. Four studies used simple tone stimuli [18,21,33,53]; two studies used speech and tone stimuli [29,54]; two studies used speech stimuli [44,57]; one study used simple tones with varying rise times [39]; and another study used a complex tone, which was developed by combining 12 pure tones [58]

#### 3.1.4. Recorded ERPs

Among the studies, only two investigated all three T-complex components (Na-Ta-Tb) [39,57]. Ta and Tb were investigated in two studies [23,54]. In other investigations, only one peak was of interest. For instance, Ta was the sole component examined in two studies [29,53], while some studies reported only the pattern of Tb [18,21]. Additionally, Na was explored in three studies [39,57,58].

Responses recorded over fronto-central sites were reported in five studies. One reported on P1 [53]; four studies reported on N1 (also called N1b) [21,23,39,58]; and one reported on P2 [39].

#### 3.1.5. Findings

Overall, the review of findings revealed abnormal T-complex responses in children with neurodevelopmental disorders. This pattern signifies neural auditory dysfunction localized in the temporal lobe across multiple neurodevelopmental disorders. Most critically, compared to the T-complex, only two studies reported atypical fronto-central responses in children with neurodevelopment disorders [21,23]. Smaller amplitudes of N1 and longer latencies of N1 were observed in children with autism [21] and LI [23] compared to those in TD, respectively. The details of the results of each study are presented in Table 1.

Regarding the T-complex, it seems that both the latency (longer and/or shorter) [21,23,54,57,58] and amplitude (smaller and/or larger) [18,29,53,55,57,58] of the T-complex showed atypical patterns. Interestingly, specific deviations were observed in various components of the T-complex across different disorders. For instance, an atypical Tb was observed in the dyslexia group, despite a normal Na and Ta [39]. Na and Tb were abnormal in another study on dyslexia [58]. The Ta component was most affected in populations with language impairment [29,55,57] and ADHD [53]. Studies on autism and DS indicated atypical Tb components [21,54]. Furthermore, findings indicated discrepancies between T-complex and fronto-central responses in studies investigating dyslexia [39,58], language impairment [23], ADHD [53], and autism [21]. There were abnormal patterns of the T-complex, despite normal fronto-central responses [39,58].

## 4. Discussion

This study reviewed the evidence of changes in T-complex and fronto-central responses in neurodevelopmental disorders. Subsequently, the findings on Na, Ta, and Tb patterns were elaborated on, as well as the different patterns observed between the T-complex and the fronto-central responses.

### 4.1. Na Pattern

Na represents the early stage of the acoustical processing of sounds [28,40,47]. In children with dyslexia, Na was larger but occurred at a similar latency compared to that in controls. This heightened response in the dyslexic population may suggest that individuals with dyslexia require greater brain activation and effort for the same early perceptual processing of auditory stimuli compared to the control group [58]. Alternatively, enhanced auditory evoked responses could be related to a failure of inhibition. Older adults normally have larger auditory evoked potentials compared to younger adults [62,63]. This age-related increased in the amplitude of the AEPs has been interpreted as a decline in the inhibitory system [64,65]. Top-down inhibition exerts a modulatory effect on sensory processing by suppressing irrelevant information from further processing [66,67]. A decreased capacity for inhibition may lead to reduced listening abilities [63,68]. This explanation may justify the larger auditory responses in children with dyslexia, as studies consistently have documented inhibitory impairments in this clinical group [69,70].

While Taylor et al. (2003) reported an effect of dyslexia on Na amplitude [58], other studies have not replicated this result [39]. Differences in stimuli complexity and experimental design may contribute to variations in the observed results across these studies. Taylor’s study employed an active paradigm involving complex stimuli composed of 12 pure tones, while Hämäläinen’s study utilized a passive paradigm featuring a single pure tone (500 Hz) with varying linear rise times [39,58]. It is therefore possible that the impact of dyslexia on Na is related to attending to complex stimuli in the study of Taylor et al., 2003. Moreover, in the study of Hämäläinen et al., 2011, despite no group effect on Na recorded in the passive condition, there was a significant effect of dyslexia on the behavioral task of discriminating stimuli with high and low rise times. In other words, the dyslexia group performed worse than the normal group when required to attend to the sound [39]. These results may suggest that the deficits lie in top-down effects, underlying neural pathways from higher centers to the auditory areas of the brain. More research and more specific study designs are warranted to differentiate between bottom-up and top-down processing in these populations.

### 4.2. Ta Pattern

The Ta component represents acoustical discrimination [28,47]. The Ta component had a smaller amplitude and longer latency in individuals with LI compared to the healthy control group [23]. A smaller Ta was consistent with three subsequent studies focusing on the same disorder [29,55,57]. Furthermore, Shafer et al. (2011) reported that the peak-to-peak amplitudes, including Na-Ta and Ta-Tb, were smaller in individuals with LI compared to healthy controls [55]. This suggests auditory discrimination impairments in people with LI. This deficit could lead to phonological processing disorders in this population [23,29]. Interestingly, the atypical pattern in people with LI was observed only with speech stimuli and not with tones, highlighting the impaired verbal processing in children with language disorders [29,57]. Similar to LI, the ADHD population exhibited a reduced Ta [53]. The atypical appearance of Ta, which indicates impaired auditory processing in the CANS, may suggest that auditory processing disorder can co-exist with ADHD [16,53,71,72].

Accordingly, reduced Ta amplitudes could reflect auditory dysfunction in ADHD and LI populations and suggest a robust association between language impairment and altered T-complex responses, particularly in the Ta component.

### 4.3. Tb Pattern

Tb is the third component of the T-complex and indexes auditory processing in secondary auditory areas [26,28,40]. Taylor et al. (2003) reported that the dyslexia group had a shorter latency of Tb compared to healthy controls [58]. This faster brain response may reflect the superficial and inappropriate processing of sound in the dyslexic population [58]. Moreover, this abnormally short latency of Tb was observed only in the right hemisphere [58]. Such findings align with neuroimaging studies reporting greater right- than left-hemisphere activity in dyslexia, which leads to faster but less accurate processing [73,74].

In children with autism, Tb was smaller and delayed compared to that in healthy controls, and the differences in Tb indexed the magnitude of the auditory impairments [18,23]. The prolonged latency of Tb may reflect slower transmission in synaptic connections of the secondary auditory areas, which may be in line with studies showing reduced blood flow in the temporal areas of this population [75,76]. Additionally, children with autism exhibited atypical inter-hemispheric differences as the intensity was modulated. Specifically, the amplitude of Tb increased more prominently in the right hemisphere than in the left hemisphere, indicating rightward lateralization. This is in contradistinction to the healthy controls, who exhibited a leftward dominance with increasing intensity [21]. This difference may reflect dysfunction in the left hemisphere and might imply the reorganization and retuning of the left and right hemispheres for amplitude processing in autism, indicating a right-hemisphere compensation for left-hemisphere dysfunction [21]. These results align with behavioral and electroencephalography (EEG) studies demonstrating a rightward lateralization of auditory processing in the autistic population [77,78].

People with Down syndrome (DS) also exhibit atypical the lateralization of auditory neural processing [54]. Specifically, Tb is delayed in people with DS. This delay is attributed to myelination deficits in this population [54]. The results also highlighted the absence of a contralateral effect on Tb. Typically, the contralateral neural pathway leads to a shorter or larger Tb response, particularly when sound is presented to the right ear, and neural activity is recorded over the left hemisphere (right ear–left hemisphere advantage of Tb) [26,36,40]. In DS, the benefit of contralateral over ipsilateral recording was not observed for Tb. These results suggest a more distributed lateralization of neural function in people with Down syndrome [54].

Lateralization is a key feature of auditory processing. Multiple studies using different techniques, including behavioral observations, functional MRI, and magnetoencephalography (MEG), have documented hemispheric lateralization at higher and lower levels of the cortex [79,80,81,82]. In other words, the functional lateralization of the brain is not limited to high-level cognitive processes like language; it starts from the lower-level neural processing of acoustical features (e.g., frequency, duration, interval, and intensity) [82,83,84]. Moreover, converging evidence illustrates that the left and right auditory cortices are asymmetrically linked to temporal and spectral processing, with the right hemisphere more sensitive to spectral features and the left to temporal features [85,86,87]. Tb may reflect the asymmetric lateralized auditory function at the level of secondary areas. Moreover, atypical development may lead to the abnormal lateralization of temporal and/or spectral processing generated in the secondary auditory cortex, which has been observed in people with autism and DS.

### 4.4. Discrepancy Between Patterns of T-Complex and Fronto-Central Responses

The second goal of this review was to compare the T-complex and fronto-central responses in children with developmental disorders. Three articles reported abnormal T-complex responses despite normal fronto-central responses [39,53,58]. Contrary to typically reduced amplitudes of P1 and N1 with a longer latency rise time, dyslexia did not show the latency effect on Tb amplitude [39]. Another study also demonstrated group effects on the amplitude of Na and the latency of Tb; the fronto-central response (N1b, a subcomponent of N1) did not show differences between TD and dyslexia groups [58]. In the case of ADHD, abnormal Ta responses were reported despite the normal P1 response [53]. The typical results of fronto-central responses have been similarly documented in individuals with APD [88,89]. However, in the aforementioned studies, only fronto-central responses are reported, so there is no such comparison between T-complex and fronto-central responses to confirm the current findings.

Among the included studies, two other studies indicated that the T-complex showed more sensitivity in tracking auditory dysfunction than the fronto-central response [21,23]. In LI, although similar to N1, the Tb latency was longer, and the latency difference recorded for Tb was higher than that for N1 (the difference for Tb was almost 20 ms; the difference for N1 was 5–10 ms) [23]. Moreover, in people with autism, N1b was smaller than in the standard group, but Tb was both smaller and more delayed compared to that in healthy controls [21]. Accordingly, Tb may be more sensitive to auditory impairments than the fronto-central response in LI and autism groups [21,23].

The divergent patterns observed between T-complex and fronto-central responses may serve to differentiate the origins of auditory deficits. The predominant generation of the T-complex occurs in the secondary auditory areas in the posterior and lateral parts of the superior temporal gyrus (Brodman 42 and 22) [35,42], while P1-N1-P2-N2 originates from the primary auditory areas in Heschl’s gyrus (Brodman 41) and the associated areas [35,90,91]. Therefore, an abnormal T-complex signifies the involvement of the secondary and associated auditory areas in a specific disorder. Additionally, different origins and patterns may suggest that the T-complex manifests different auditory processes that are distinct from those reflected in fronto-central responses. For instance, Tb could index abnormal processing related to varying rise time and intensity, even in the presence of normal fronto-central responses, as well as reflecting the hemispheric lateralization of auditory processing at secondary auditory areas.

### 4.5. Limitations and Future Direction

Although T-complex differences were related to auditory dysfunction in all experimental groups, the patterns of all components of the T-complex were not reported. Some studies investigated only one component. It is unclear if other components would show the same pattern. For instance, the study of Shafer in 2011 investigated only Ta. Na and Tb were the only components of interest in the studies of Hämäläinen et al., 2011, and Bruneau et al., 1999, respectively. More research investigating all T-complex components is warranted to confirm the results.

Also, fewer than half of the studies reported and compared both fronto-central and temporal responses. It is not clear whether the conflicting results between these two AEPs could be confirmed in other disorders. Moreover, no study has investigated and compared these responses in APD. Similar to the results of fronto-central responses in dyslexia and ADHD discussed in this review, the literature has indicated that AEPs recorded at fronto-central sites did not consistently track auditory impairments in APD groups. One reason may lie in the fact that the related studies on APD have not assessed the T-complex. Hence, researching the effect of APD on the T-complex and comparing it with the fronto-central response using different methodologies may advance the knowledge about the origins of the dysfunction in APD and introduce a consistent biomarker for APD.

## 5. Conclusions

This study revealed that the T-complex can index auditory processing impairments at the cortical level in various neurodevelopmental disorders, even when there is a normal pattern of the fronto-central response. These opposite patterns may emphasize the different generators between T-complex and fronto-central responses.

Also, these findings may suggest that the neural generators of the T-complex contribute to distinct auditory processes that differ from those indexed by the fronto-central response and neural generators. Atypical T-complex patterns in all investigated experimental groups may also highlight the potential of the T-complex as a sensitive biomarker for identifying auditory processing abnormalities across a range of neurodevelopmental conditions.

## Figures and Tables

**Table 1:** (**A**) Summary of included studies. (**B**) Summary of results.

**A**					
**Authors**	**Year**	**Experimental Group**	**Control Group**	**Size and Age**	**AEPs**
Bishop, Hardiman, and Barry [29]	2012	Specific language impairment group	Normal children and teenagers	16 children (7–11) and 16 teenagers (12–16)	Ta
Bruneau, Roux, Adrien, and Barthélémy [21]	1999	Autism group and intellectual disability group	Normal children	16 autistic children (4–8) 16 intellectually disabled children (4–8) (4–8)	N1b, N1c (Tb)
Bruneau, Bonnet-Brilhault, Gomot, Adrien, and Barthélémy [18]	2003	Autism group	Normal children	Autism: 16 children (4–8) Normal: 26 children (4–8)	N1c (Tb)
Gomes, Duff, Ramos, Molholm, Foxe, and Halperin [53]	2012	ADHD group	Normal adults and children	ADHD: 15 children (7.3–12.5) Normal: 16 adults (18–45) and 15 children (8 7.5–13.5)	P1, Ta
Groen, Alku, and Bishop [54]	2008	Down syndrome group	Normal children	DS: 19 children (10–12). Normal: 19 children (10–12)	Ta-Tb
Hämäläinen, Fosker, Szücs, and Goswami [39]	2011	Dyslexia group	Normal adults	11 adults (20–54)	N1-P2, Na-Ta-Tb
Rinker [57]	2021	Developmental language disorder, monolingual German children, and bilingual Turkish–German children	Normal monolingual and bilingual children	14 monolingual German children with DLD (6 49–73 months) 16 monolingual German children (4.2–6) 12 Turkish–German children (55–81 months)	Na-Ta-Tb
Shafer [55]	2011	Specific language impairment	Normal children	22 SLI children 32 normal children	Na-Ta-Tb
Taylor [58]	2003	Dyslexia group	Normal children	DS: 11 children (average age = 10.08) Normal: 11 children (average age = 10.29±)	N1a (Na)-N1b-N1c (Tb)
Tonnquist-Uhlen [23]	1996	Severe language impairment group	Normal children	Normal: 20 children (9–15) SLI: 20 children (9–15)	Ta-Tb, N1
**B**					
**Author**	**Year**	**Reported Measurements**	**Results**		
			**Results Related to the T-Complex** **(Na-Ta-Tb), Amplitude**	**Results Related to the T-Complex** **(Na-Ta-Tb), Latency**	**Results Related to the Fronto-Central Response (P1-N1-P2)**
Bishop, Hardiman, and Barry [29]	2012	Amplitude of Ta	Ta in SLI was smaller (*p* < 0.001) than Ta in TD. This difference was not significant with tone stimuli (*p* = 0.1) but highly significant with speech stimuli (*p* = 0.002).	-	Not measured
Bruneau, Roux, Adrien, and Barthélémy [21]	1999	Amplitude and latency of N1a, N1b, and N1c	The amplitude of N1c was smaller for AUT than for RET and normal (*p* < 0.0001). N1b and N1c showed longer and smaller amplitudes among AUT compared to normal children (*p* < 0.0001).	The longest latency of N1c was seen for AUT. RET and normal did not differ in N1c latency. N1b and N1c showed longer latency among AUT compared to normal children (*p* < 0.0001).	The smallest and longest N1b was observed in the RET group (*p* < 0.02). There was a smaller N1b in AUT than in the normal group (*p* < 0.0001). The latency of N1b was not different between normal and AUT groups.
Bruneau, Bonnet-Brilhault, Gomot, Adrien, and Barthélémy [18]	2003	Amplitude and latency of Tb	A smaller amplitude of Tb was seen in AUT than in normal (*p* < 0.0005 and *p* < 0.02, respectively).	A longer latency of Tb was seen in AUT than in normal (*p* < 0.0005 and *p* < 0.02, respectively).	
Gomes, Duff, Ramos, Molholm, Foxe, and Halperin [53]	2012	Amplitude of Ta and P1	Ta had a smaller amplitude (*p* < 0.005) in ADHD for attended and unattended conditions.	-	There was no difference in the P1 peak between ADHD and normal groups.
Groen, Alku, and Bishop [54]	2008	Amplitude and latency of Ta and Tb	A larger contralateral Tb was seen in typical children (*p* = 0.01), not in DS (*p* = 0.5).	A shorter contralateral Ta was seen at T7 in typical children (*p* = 0.00) but not in DS children (*p* = 0.5). Tb was 12 ms delayed in the DS group compared to the normal group (*p* = 0.001).	Not measured
Hämäläinen, Fosker, Szücs, and Goswami [39]	2011	Amplitude and latency of Na, amplitude of Ta and Tb, amplitude and latency of N1 and P2	No group difference was seen based on the Na peak. The amplitude of Na did not show the effect of rise time (RT) changes. Only the Tb wave of the T-complex showed a group difference as a function of sound rise time. Participants with dyslexia did not show a similar reduction pattern in Tb amplitude to the normal group with increasing rise times (*p* < 0.005).	-	N1 and P2 showed typical morphology in adults with and without dyslexia. The changes in N1 and P2 as a function of rise time were typical.
Rinker [57]	2021	Latency of Na and Tb, amplitude of Ta	The difference between DLD and TD was significant only with vowel stimuli. The group effect was dependent on the stimuli type for Ta; for vowel stimuli, Ta was smaller for DLD and bilingual than TD (*p* = 0.04). In tone condition, Ta was smaller (*p* < 0.05) for bilingual than for DLD and TD (DLD and TD did not differ).	Regarding Na, bilingual had a longer latency than TD and DLD (*p* = 0.01). Tb had a shorter latency (*p* < 0.001) for DLD than TD and bilingual (bilingual and TD did not differ).	Not measured
Shafer [55]	2011	Amplitude of Ta and peak-to-peak amplitude of Na-Ta and Ta-Tb	Ta was smaller in the LI group than in the normal group (*p* < 0.05). The peak-to-peak amplitudes, including Na-Ta and Ta-Tb, were also smaller in the LI group than in the normal group (*p* < 0.05).	-	Not measured
Taylor [58]	2003	Amplitude and latency of N1a, N1b, and N1c	N1a was larger in dyslexia than in the normal group. The N1c amplitude was larger for mistuned than tuned stimuli (*p* < 0.025), with no group effects or interactions.	There was an interaction with stimuli: When the stimuli were tuned (*p* < 0.018), N1a was shorter for dyslexia than normal, and vice versa for mistuned stimuli (*p* < 0.02). N1c was shorter for the dyslexic than control children (*p* < 0.029) (with no interaction with stimuli), but only over the right lateral temporal electrode.	N1b did not show a group difference between normal and dyslexia groups. This response was larger for mistuned stimuli.
Tonnquist-Uhlen [23]	1996	Amplitude and latency of Tb, amplitude of Ta	A smaller amplitude of Tb was reported in LI children, but the results did not reach significance.	Ta was longer in LI children (*p* < 0.001). Tb was longer in the LI group than normal (*p* < 0.0005–0.001). In both groups, there was a shorter Tb at T7, regardless of the ear.	N1 was prolonged in the LI group. However, the difference om N1 latency between the normal and experimental groups was not as high as the difference in Tb latency. The difference for Tb was almost 20 ms, and the difference for N1 was 5–10 ms

AEP: auditory event-related potential, ADHD: attention disorder/hyperactivity, AUT: autism with intellectual disability, DLD: developmental language disorder, DS: Down syndrome, LI: language impairment, RET: intellectual disability without autism, SLI: specific language impairment.

## Abbreviations

Attention deficit/hyperactivity disorder (ADHD), auditory evoked potentials (AEPs), auditory processing disorder (APD), autism with intellectual disability (AUT), central auditory neural system (CANS), developmental language disorder (DLD), Down syndrome (DS), Heschl’s gyrus (HG), interstimulus interval (ISI), language impairment (LI), medial geniculate body (MGB), primary auditory cortex (PAC), planum polare (PP), planum temporale (PT), intellectual disability without autism (RET), specific language impairment (SLI), stimulus onset asynchrony (SOA), superior temporal gyrus (STG), typically developing (TD).

## Appendix A. Inclusion and Exclusion Criteria Applied to the Scoping Review

**Table A1:** Inclusion and exclusion criteria.

	Inclusion Criteria	Exclusion Criteria
Population (age and condition)	Children or adults, normal and neurodevelopmental disorders such as attention deficit/hyperactivity disorder, language impairment, and autism	The participants were in general health and the study did not include clinical groups
Evaluation	T-complex	Subcortical responses (ABR), ASSR, and other late auditory responses such as MMN, P300, and N400
Publication type	Peer-reviewed journals and gray literature published after 1950 and only in English	Any unscientific papers, magazines, editorials, and manuals or text in a language other than English
Outcome	Peak and latency of Na-Ta-Tb or Ta-Tb	No results related to T-complex components

## Appendix B. Recorded Parameters of Stimuli and ERPs

**Table A2:** The stimuli and recording features of included studies.

	Authors	Year	Title	Type (Single or Paired, Tone, Speech, or Complex Tone (Composed of Multiple Tones))	Intensity	Transducer	Delivery and Recording	ISI or SOA	Reference	Sample Rate	Filter
1	Bishop, Hardiman, and Barry	2012	Auditory Deficit as a Consequence Rather than Endophenotype of Specific Language Impairment: Electrophysiological Evidence	Single, 1000 Hz and /bah/	86.5 dB SPL	Headphone	Monaural; the study did not mention the mode of stimulation	SOA, 1 s	Mastoid right or left	500 Hz	0.1–70 Hz
2	Bruneau, Roux, Adrien, and Barthélémy	1999	Auditory associative cortex dysfunction in children with autism: evidence from late auditory evoked potentials (N1 wave ± T-complex)	Single, 750 Hz tone burst	50, 60, 70, and 80 dB SPL	Two speakers	Binaural	ISI, from 3 to 5 s	Linked earlobes	250 Hz	NMA
3	Bruneau, Bonnet-Brilhault, Gomot, Adrien, and Barthélémy	2003	Cortical auditory processing and communication in children with autism: electrophysiological/behavioral relations	Single, 750 Hz tone	50, 60, 70, and 80 dB SPL	Two speakers	Binaural	ISI, 3 to 5 s	Linked earlobes	250 Hz	NMA
4	Gomes, Duff, Ramos, Molholm, Foxe, and Halperin	2012	Auditory selective attention and processing in children with attention deficit/hyperactivity disorder.	Single, four types of two standards [1000 Hz] (low channel) and two [2000 Hz] (high channel)	82 dB SPL	Insert earphones	Monaural; the study did not mention the mode of stimulation	SOA, 850 to 1150 ms	Tip of the nose	0.5–70 Hz	500 Hz
5	Groen, Alku, and Bishop	2008	Lateralisation of auditory processing in Down syndrome: A study of T-complex peaks Ta and Tb.	Single, vowel /a/, 576 Hz for the simple tone; the complex tone was composed of four tones of 576, 1055, 2589, and 3163 Hz	50 dB SPL	Insert earphones	Monaural, ipsi and contra conditions	ISI, 1550 ms	left mastoid	1000 Hz	0.1–70 Hz.
6	Hämäläinen, Fosker, Szücs, and Goswami	2011	N1, P2 and T-complex of the auditory brain event-related potentials to tones with varying rise times in adults with and without dyslexia.	Single, 500 Hz with varying linear rise times: 10, 30, 60, 90, and 120 ms	75 dB SPL	Insert headphones	Binaural	ISI, 2.5–3.5 s	Vertex	500 Hz	0.1–200 Hz
7	Rinker	2021	Language Learning Under Varied Conditions: Neural Indices of Speech Perception in Bilingual Turkish-German Children and in Monolingual Children With Developmental Language Disorder (DLD)	Single, vowel [ε]	90 dB SPL	Headphones	Binaural	ISI, 650 ms	Left earlobe	500 Hz	0.1–70 Hz
8	Shafer	2011	Evidence of deficient central speech processing in children with specific language impairment: The T-complex	Various stimuli: “the” repetition standard /E/ standard /E/ first word in a pair of words.	65 dB SPL, 86.5 dB SPL, 86.5 dB SPL, 70 dB SPL	Insert phone	NM	ISI, 625 ms, 550 ms, 350 ms, 2000 ms	Nose	512 Hz	0.05–100 Hz
9	Taylor	2003	Neurophysiological Measures and Developmental Dyslexia: Auditory Segregation Analysis	Five complex sounds were obtained by combining 12 pure tones. Four of the stimuli were mistuned, and their third harmonic was increased by 2, 3, 8, or 16% of original values.	70 dB SPL	Headphones	Binaural	ISI, 1.1 s	Cz	500 Hz	0.1–30 Hz
10	Tonnquist-Uhlen	1996	Topography of Auditory Evoked Long-Latency Potentials in Children with language impairment	Single, 500 Hz	75 dB HL	Insert phone	Monaural, ipsi and contra conditions	ISI, 1.0 s	Chin	500 Hz	0.1–60 Hz

dB = decibel, SL = sound level, nHL = normal hearing level, SPL = sound pressure level, Hz = Hertz, ipsi = ipsilateral, contra = contralateral, NM = not mentioned, N = number of articles, ISI = interstimulus interval, SOA = stimulus onset asynchrony, s = second, ms = millisecond.
